# Relationship of subjective and objective sleep measures with physical performance in advanced-stage lung cancer patients

**DOI:** 10.1038/s41598-021-96481-7

**Published:** 2021-08-26

**Authors:** Naomi Takemura, Denise Shuk Ting Cheung, Daniel Yee Tak Fong, Anne Wing Mui Lee, Tai-Chung Lam, James Chung-Man Ho, Tsz Yeung Kam, Jeannie Yin Kwan Chik, Chia-Chin Lin

**Affiliations:** 1 School of Nursing, Li Ka Shing Faculty of Medicine, The University of Hong Kong, 4/F, William M.W. Mong Block, 21 Sassoon Road, Pokfulam, Hong Kong; 2 Department of Clinical Oncology, The University of Hong Kong-Shenzhen Hospital, Guangdong, China; 3 Department of Clinical Oncology, Li Ka Shing Faculty of Medicine, The University of Hong Kong, Pokfulam, Hong Kong; 4 Department of Medicine, Li Ka Shing Faculty of Medicine, The University of Hong Kong, Pokfulam, Hong Kong; 5 Department of Clinical Oncology, Pamela Youde Nethersole Eastern Hospital, Chai Wan, Hong Kong; 6 Department of Clinical Oncology, Queen Elizabeth Hospital, Kowloon, Hong Kong; 7 School of Nursing, College of Nursing, Taipei Medical University, Taipei, Taiwan; 8 Alice Ho Miu Ling Nethersole Charity Foundation Professor in Nursing, Kowloon, Hong Kong

**Keywords:** Oncology, Risk factors

## Abstract

Advanced lung cancer patients suffer from deteriorated physical function, which negatively impacts physical and psychological health. As little is known about sleep and physical function in this population, this study aimed to examine the association between subjective and objective sleep parameters and physical function among them. 164 advanced lung cancer patients were included. Objective sleep was measured by actigraphy (measured on non-dominant wrist for 72 h), and subjective sleep quality was assessed by the Pittsburgh Sleep Quality Index (PSQI). Performance-based physical function was measured by Timed Up and Go Test (TUGT), 6-Minute Walk Test (6MWT), Sit-to-Stand Test, and One-leg Standing Test. Univariable and multivariable regression analyses were employed to examine the association between sleep and physical function. Total sleep time (TST) was significantly associated with the 6MWT (β = 0.259; 95% CI 0.120, 0.398; P < 0.001), TUGT (β = − 0.012; 95% CI = − 0.017, − 0.008; P < 0.001) and Sit-to-Stand Test (β = 0.027; 95% CI = 0.018, 0.035; P < 0.001) after adjustment for multiple covariates. PSQI global score was only significantly associated with TUGT (β = 0.140; 95% CI = 0.000, 0.280; P = 0.050) after adjustment for multiple covariates. Shorter sleep duration significantly predicted poorer physical performance in advanced lung cancer patients, and more attention is required for those with less than 4.3 h of sleep on average.

Trial registration: ClinicalTrials.gov, NCT03482323. Registered 29 March 2018, https://clinicaltrials.gov/ct2/show/NCT03482323; ClinicalTrials.gov, NCT04119778. Registered 8 October 2019, https://clinicaltrials.gov/ct2/show/NCT04119778.

## Introduction

Lung cancer is one of the most common cancers and causes of cancer-related deaths worldwide^1^. A substantial portion (75%) of lung cancer patients are diagnosed at an advanced stage^2^. The majority of advanced cancer patients suffer from deteriorating physical function due to disease progression and cancer treatment^3^. Lung cancer patients report lower physical function than patients affected by other cancer types^4^. Impaired physical function reduces patient autonomy, compromises quality of life, and jeopardizes overall cancer survival^5,6^. Furthermore, functional impairment and decline escalate the distress level and add to the disease burden of patients and their families^6^. Thus, identifying factors that contribute to a decline in physical function in advanced lung cancer patients is crucial to prevent or postpone the disablement process.

Factors associated with physical function in advanced cancer patients include fatigue and performance status^7^. Sleep is another potential factor related to physical function that deserves further investigation in cancer populations. Several studies conducted among athletes demonstrated that sleep deprivation negatively impairs their physical performance and increases their reaction time and perceived exertion during exercise^8,9^. There is also emerging evidence in community-dwelling elderly individuals that self-reported sleep disturbances, together with objectively measured poor sleep, are associated with slower walking speed, weaker muscle strength, and functional performance impairment^10–12^. For cancer populations, two studies showed that patients with insomnia had more physical impairment^13,14^. However, both studies adopted self-report sleep questions that were subject to reporting bias^13,14^. Additionally, only one study used a validated physical performance test (Short Physical Performance Battery)^14^, while another used a self-report questionnaire^13^.

There has been no research examining the association between sleep and physical functions in cancer populations utilizing both subjective and objective sleep measures and validated physical performance tests. Among different cancer types, lung cancer was found to be associated with poor physical function and functional decline over time in patients^15^. Additionally, patients with lung cancer had either the highest or second-highest level of sleep problems compared to other cancer populations^15,16^. Therefore, the present study aimed to examine the association between subjective and objective measures of sleep parameters and performance-based measures of physical function in advanced lung cancer patients. It is hypothesized that both subjective and objective sleep parameters predict physical performance in this population. Our findings shed light on whether sleep predicts physical performance in an advanced lung cancer population. Such information will guide sleep assessment in cancer patients for predicting physical function and related outcomes, including quality of life and survival.

## Methods

### Participants

This study reports a subset of baseline data from a randomized controlled trial examining the effect of exercise in patients with advanced-stage lung cancer. The trial was registered with ClinicalTrials.gov (identifier: NCT03482323 registered on 29/3/2018) and (identifier: NCT04119778 registered on 8/10/2019). Patients were eligible if they were (1) diagnosed with stage IIIB or IV non-small cell lung cancer; (2) of 0–2 Eastern Cooperative Oncology Group Performance Status; (3) not diagnosed with other cancer a year before; (4) not exercising regularly (defined as < 150 min of moderate-intensity exercise per week) in daily living; and (5) not participated in current research studies or other aerobic exercise or mind–body exercises. Patients were excluded from the study if they were suffering from a clinically diagnosed neurological, or psychiatric disorder and had not completed the questionnaires or physical functioning tests.

### Procedures

Patients were approached by research personnel and recruited from outpatient clinics at three hospitals in Hong Kong from May 2018 to Jan 2020. Written informed consent was obtained from all patients before questionnaires and functioning tests commenced. At study entry, patients completed the questionnaires and physical functioning tests conducted by research personnel following standard protocols^17–20^. Additionally, patients had to wear an actigraph on their non-dominant wrist for 72 h. The study was approved by the Institutional Review Board of the University of Hong Kong/Hospital Authority Hong Kong West Cluster, Hong Kong East Cluster, and Kowloon Central Cluster/Kowloon East Cluster.

### Sleep measures

#### Subjective sleep quality

Subjective sleep quality was assessed by the Pittsburgh Sleep Quality Index (PSQI)^21^. The PSQI is a self-completed, validated questionnaire that assesses sleep quantity and quality during the past month. It consists of 19 questions that encompass seven dimensions: subjective sleep quality, sleep latency, sleep duration, habitual sleep efficiency, sleep disturbance, sleep medication usage, and daytime dysfunction^21^, and thus provides a relatively more detailed assessment of sleep quality than other sleep measures. Each dimension score ranges from 0 to 3, rendering a total score of 0 to 21 in which score level negatively is correlated with sleep quality. A total score > 5 denotes poor sleep^21^. The PSQI has been validated in the Chinese population^22^. In addition, the PSQI has been found to be a reliable and valid measure of sleep in cancer patients^23^, and is the most commonly used tool to assess sleep quality in cancer patients in two recently published reviews^24,25^.

#### Objective sleep parameters

Sleep was also assessed objectively by using wrist actigraphy (Actigraph; Ambulatory Monitoring Inc., New York). An actigraph was worn on the non-dominant wrist for 72 h. Participants were asked to complete a sleep diary for the duration of time they wore the actigraph which was used to revise the actigraphy data.

Sleep parameters measured by actigraphy included the following: (1) total sleep time (TST), defined as the hours per night spent sleeping while in bed; (2) sleep efficiency, calculated as TST divided by the time between bedtime and rise-time multiplied by 100; (3) wake after sleep onset, measured as the sum of all wake epochs during the sleep period (reflecting the number of minutes that exceeded the sensitivity threshold and were scored as awake); and (4) the movement and fragmentation index (MFI), the number of interruptions of sleep by physical movement, calculated as the number of groups of consecutive mobile 20-s epochs divided by the total number of immobile epochs multiplied by 100. The MFI captures all movements regardless of the intensity of the movement^26^. Actigraphy data for each patient were averaged over a 24-period to reduce night-to-night variability.

### Physical functioning tests

#### Timed up and go (TUGT)

The Timed Up and Go Test is a test of balance that is commonly used to examine an individual’s functional mobility^17^. It measures the time an individual needs to stand up from a standard armchair, turn, walk back, and sit down. The time taken to complete the test is strongly correlated with the level of functional mobility. This is a reliable and valid test for quantifying functional mobility, which is a term used to reflect the balance and gait manoeuvres used in everyday life (e.g., getting in and out of the chair, walking, and turning)^17^.

#### Sit-to-stand test

The sit-to-stand test is used to measure an individual’s lower limb strength, and it measures the number of repetitions an individual has completed in a given period from a chair^18^. An individual is instructed to have their hands folded in front of the chest with their feet flat on the floor. The test has been reported to be associated with standing, leaning balance^27^, and mobility^27^.

#### 6-minute walk test

The 6-min walk test (6MWT) measures the distance an individual can walk at a constant, uninterrupted, and unhurried pace in 6 min^20^. It is a simple and inexpensive method for assessing exercise capacity at a submaximal level. All 6MWTs were conducted using a lap 20–25 m in length on flat, hard ground, according to the American Thoracic Society guidelines^28^. Walk tests were timed with a stopwatch. The number of full laps completed was counted, and the distance covered in the last lap was determined; hence, the total distance in metres was calculated for each walk.

#### One-leg standing test

One-leg standing test measures the time one can stand on one lower limb without support^19^. This test is a clinical tool that assesses postural steadiness in a static position by quantitative measurement^29^. An individual is asked to stand initially in a relaxed stance with their weight evenly distributed between both legs. Without using any assistive device, he/she is instructed to stand on the leg they select while keeping their arms by their sides. Their eyes remain open during the test.

### Covariates

Predisposing factors that were known to affect both sleep and physical function in cancer patients encompassing background characteristics, psychological distress, fatigue, and daily physical activity level were measured^7,30–33^.

#### Background characteristics

Sociodemographic variables, cancer-related information, and lifestyle factors were collected via a self-designed questionnaire. Sociodemographic variables included age, gender, marital status, and education level. Cancer-related information comprised current treatment modalities (chemotherapy or nonchemotherapy), time since diagnosis, and lifestyle factors consisting of smoking (smoker or nonsmoker) and drinking (drinker or nondrinker) habits. Body mass index (BMI) was calculated as weight in kilograms divided by height in metres squared. The Karnofsky Performance Status (KPS) score, which measures the level of patient activity and patient independence, was assessed by nurses^34^.

#### Psychological distress

Psychological distress (i.e., anxiety and depression symptoms) was measured using the Chinese version of the Hospital Anxiety and Depression Score (HADS), with a score of 8 or more on either subscale representing clinical cases of anxiety or depression^35^.

#### Fatigue

Fatigue was assessed using the Chinese version of the Brief Fatigue Inventory (BFI), comprising 9 items with each scored on a 0–10 numeric scale^36^. A higher score indicates a higher level of fatigue.

#### Daily physical activity level

Daily physical activity level (i.e., step count) was measured via actigraphy.

#### Charlson comorbidity index (CCI)

The Charlson Comorbidity Index was developed to estimate the 1-year mortality risk and disease burden^37^. The CCI takes into account 19 comorbid conditions with each comorbidity weighted 1, 2, 3, or 6 for its relative risk of 1-year mortality^37^. The CCI has demonstrated excellent predictive validity for numerous clinical outcomes and a number of malignancies^38^.

### Statistical analysis

Descriptive statistics were used to describe the sociodemographic, clinical, and treatment characteristics of the patient population. Characteristics were summarized as the mean ± standard deviation (SD) for continuous variables and counts and percentages for categorical variables.

Regression analysis was employed to estimate the association between subjective (PSQI global score and its seven components) and objective sleep measures (actigraphy, i.e. total sleep time, sleep efficiency, and wake after sleep onset) and physical functioning tests (6-Minute Walk Test, One-leg Stand Test, Timed Up and Go Test, and Sit-to-Stand Test). Univariable regression analysis was first performed to examine the association of a single sleep measure with each physical functioning test. Subsequently, multivariable regression analysis was performed with covariates added to the linear model. The covariates added included age, gender, BMI, education, time since diagnosis, current treatment, marital status, KPS score, step count per day, smoking, drinking, fatigue, anxiety, depression, and CCI. For both models, data were checked for a linear relationship, absence of multicollinearity, homoscedasticity, and a normal distribution of residuals.

A receiver operating characteristic (ROC) curve analysis was conducted to determine a cut-off value for each sleep parameter that best identified individuals with poor physical performance. Specifically, it was taken as the one that maximized the Youden index, that is, sensitivity + specificity−1. The accuracy and utility of the cut-off were assessed by the sensitivity, specificity, and positive and negative predictive values.

All analyses were conducted with the Statistical Package for Social Sciences (SPSS, v.25.0, IBM SPSS Statistics, IBM Corporation) and were 2-sided. A P value of less than 0.05 was regarded statistically significant.

### Ethics approval and consent to participate

The study was performed in accordance with the Declaration of Helsinki. Ethics approval was granted by the Institutional Review Board of the University of Hong Kong/Hospital Authority Hong Kong West Cluster (Ref: UW 18-154), Hong Kong East Cluster (Ref: HKECREC-2019-014), and Kowloon Central Cluster/Kowloon East Cluster (Ref: KC/KE-19-0039/ER-3). The participants all consented to participate in the study.

## Results

### Subject characteristics

A total of 180 patients aged 35 to 81 years enrolled in the study from 2018 to 2020, and 164 patients with complete data were included in the analysis. Data were collected via questionnaires, objective physical performance tests, and actigraphy. Their baseline characteristics are listed in Table 1. The mean age of the included patients was 61.16 ± 8.80. Patients, in general, were not classified as being clinically anxious or depressed. Slightly less than half (49.4%) of patients were classified as poor sleepers by the PSQI questionnaire. Their sleep duration, on average, was 4.70 ± 1.50 h, while their mean sleep efficiency was 91.70 ± 3.80%.

**Table 1:** Characteristics of the Study Sample (*N* = 180).

Sociodemographic characteristics	Mean (*SD*)	*n* (%)
Age (years)	61.16 (8.80)	
**Sex**		
Male		85 (47.2%)
Female		95 (52.8%)
BMI, kg/m^2^	22.31 (3.47)	
KPS score	90.22 (7.17)	
**Marital status**		
Single/divorced/widowed		37 (20.6%)
Married/cohabiting		143 (79.4%)
**Active smoker**		
No		170 (94.4%)
Yes		10 (5.6%)
**Active drinker**		
No		170 (94.4%)
Yes		10 (5.6%)
**Current treatment modalities**		
Chemotherapy		55 (30.6%)
Non-chemotherapy		125 (69.4%)
Targeted therapy		101 (80.8%)
Radiotherapy		2 (1.6%)
Immunotherapy		14 (11.2%)
No treatment		8 (6.4%)
**Sleep quality**		
Poor (PSQI > 5)		89 (49.4%)
Good (PSQI ≤ 5)		91 (50.6%)
Sleep duration, hour	4.70 (1.50)	
Sleep efficiency, %	91.70 (3.80)	
Charlson Comorbidity Index	6.40 (0.71)	
Depression (HADS)	5.56 (3.60)	
Anxiety (HADS)	4.87 (3.36)	
Fatigue (BFI)	2.39 (2.07)	
6MWT, m	403.18 (88.82)	
TUGT, s	8.77 (2.96)	
Sit-to-Stand Test, n	8.21 (5.29)	
OLS Test, s	54.41(85.15)	

*BFI* Brief Fatigue Inventory, *BMI* body mass index, *HADS* Hospital Anxiety and Depression Score, *KPS* Karnofsky Performance Status, *PSQI* Pittsburgh Sleep Quality Index, *6MWT* Six-minute Walking Test, *TUGT* timed up and go test, *OLS Test* One-leg Standing Test, *SD* standard deviation.

### Subjective sleep measures and physical performance

Tables 2 and 3 display the univariable and multivariable regression models, respectively, for subjective sleep measures (PSQI and its seven components).

**Table 2:** Univariable regression model for subjective sleep measures with physical performance.

	PSQI Global Score		Subjective sleep quality		Sleep latency		Sleep duration		Habitual sleep efficiency	
	Beta (95% CI)	*p*	Beta (95% CI)	*p*	Beta (95% CI)	*p*	Beta (95% CI)	*p*	Beta (95% CI)	*p*
Physical functioning tests										
6MWT	− 2.032 (− 6.086, 2.023)	0.324	− 4.902 (− 19.713, 9.910)	0.514	5.028 (− 8.984, 19.040)	0.480	− 12.004 (− 24.628, 0.620)	0.062	− 6.564 (− 17.103, 3.975)	0.220
TUGT	0.205 (0.067, 0.344)	0.004*	0.074 (− 0.443, 0.592)	0.777	0.177 (− 0.312, 0.666)	0.475	0.941 (0.521, 1.362)	< 0.001*	0.653 (0.298, 1.008)	< 0.001*
Sit-to-stand test	− 0.179 (− 0.432, 0.075)	0.166	− 0.015 (− 0.945, 0.915)	0.975	− 0.708 (− 1.581, 0.166)	0.112	− 0.856 (− 1.645, − 0.066)	0.034*	− 0.211 (− 0.874, 0.453)	0.532
One-leg standing test	− 3.809 (− 7.954, 0.337)	0.072	− 5.199 (− 20.449, 10.051)	0.502	− 5.240 (− 19.668,9.188)	0.474	− 16.588 (− 29.474, − 3.702)	0.012*	− 7.987 (− 18.819, 2.845)	0.147

	Sleep disturbances		Use of sleep medication		Daytime dysfunction					
	Beta (95% CI)	*p*	Beta (95% CI)	*p*	Beta (95% CI)	*p*				
Physical functioning tests										
6MWT	− 22.762 (− 46.661, 1.137)	0.062	9.659 (− 8.516, 27.835)	0.296	5.476 (− 12.932, 23.884)	0.558				
TUGT	1.464 (0.653, 2.276)	0.001*	− 0.528 (− 1.159, 0.103)	0.100	− 0.459 (− 1.098, 0.180)	0.158				
Sit-to-stand test	− 1.053 (− 2.560, 0.454)	0.170	0.518 (− 0.624, 1.659)	0.372	0.080 (− 1.076, 1.235)	0.892				
One-leg standing test	− 24.269 (− 48.859, 0.321)	0.053	6.967 (− 11.782, 25.715)	0.464	4.654 (− 14.307, 23.615)	0.629				

*p < 0.05.

**Table 3:** Multivariable regression model for subjective sleep measures with physical performance.

	PSQI Global Score^†^		Subjective sleep quality^†^		Sleep latency^†^		Sleep duration^†^		Habitual sleep efficiency^†^	
	Beta (95% CI)	*p*	Beta (95% CI)	*p*	Beta (95% CI)	*p*	Beta (95% CI)	*p*	Beta (95% CI)	*p*
Physical functioning tests										
6MWT	0.786 (− 3.451, 5.023)	0.715	4.795 (− 10.178, 19.767)	0.528	8.000 (− 5.882, 21.881)	0.257	− 12.188 (− 24.811, 0.434)	0.058	0.425 (− 9.880, 10.731)	0.935
TUGT	0.140 (0.000, 0.280)	0.050*	− 0.018 (− 0.520, 0.483)	0.943	0.126 (− 0.340, 0.592)	0.593	0.849 (0.445, 1.253)	< 0.001*	0.440 (0.103, 0.777)	0.011*
Sit-to-stand test	− 0.157 (− 0.444, 0.129)	0.279	0.178 (− 0.839, 1.195)	0.730	− 0.831 (− 1.767, 0.106)	0.082	− 0.787 (− 1.645, 0.071)	0.072	0.033 (− 0.666, 0.732)	0.926
One-leg standing test	− 3.678 (− 8.047, 0.691)	0.098	− 6.006 (− 21.574, 9.562)	0.447	− 8.584 (− 23.022, 5.855)	0.242	− 12.856 (− 25.984, 0.272)	0.055	− 5.098 (− 15.788, 5.591)	0.347

	Sleep disturbances^†^		Use of sleep medication^†^		Daytime dysfunction^†^					
	Beta (95% CI)	*p*	Beta (95% CI)	*p*	Beta (95% CI)	*p*				
Physical functioning tests										
6MWT	0.034 (− 26.323, 26.390)	0.998	9.608 (− 8.551, 27.767)	0.297	7.959 (− 10.636, 26.555)	0.399				
TUGT	0.984 (0.117, 1.850)	0.026*	− 0.596 (− 1.198, 0.006)	0.052	− 0.609 (− 1.224, 0.007)	0.052				
Sit-to-stand test	− 0.661 (− 2.447, 1.124)	0.465	0.171 (− 1.065, 1.408)	0.785	− 0.117 (− 1.382, 1.148)	0.855				
One-leg standing test	− 12.817 (− 40.159, 14.524)	0.356	0.830 (− 18.133, 19.793)	0.931	3.088 (− 16.299, 22.476)	0.753				

*p < 0.05.

^**†**^Adjusted for age, gender, BMI, education level, time since diagnosis, current treatment, marital status, KPS score, step count, smoking, drinking behaviour, fatigue, anxiety, depression and Charlson Comorbidity Index.

The PSQI global score was significantly associated with TUGT (β = 0.205; 95% CI 0.067, 0.344; P = 0.004). Regarding subscales, sleep disturbances (β = 1.464; 95% CI 0.653, 2.276; P = 0.001) and habitual sleep efficiency (β = 0.653; 95% CI 0.298, 1.008; P < 0.001) were only significantly associated with TUGT. Sleep duration was significantly associated with TUGT (β = 0.941; 95% CI 0.521, 1.362; P < 0.001), Sit-to-Stand Test (β = − 0.856; 95% CI 1.645, − 0.066; P = 0.034) and One-leg Standing Test (β = − 16.588; 95% CI − 29.474, − 3.702; P = 0.012).

After adjustment of covariates (i.e., background characteristics, psychological distress, fatigue, daily physical activity level, and CCI), the significant association between PSQI global score (β = 0.140; 95% CI 0.000, 0.280; P = 0.050), sleep duration (β = 0.849; 95% CI 0.445, 1.253; P < 0.001), habitual sleep efficiency (β = 0.440; 95% CI 0.103, 0.777; P = 0.011), sleep disturbances (β = 0.984; 95% CI 0.117, 1.850; P = 0.026), and TUGT remained, but significant associations with other physical functioning tests did not. The variance inflation factor (VIF) for all factors was below 5, indicating the absence of multicollinearity problems.

### Objective sleep measures and physical performance

Tables 4 and 5 show the univariable and multivariable regression models, respectively, for objective sleep measures (sleep duration, sleep efficiency, and wake after sleep onset) as measured by actigraphy. Sleep duration significantly predicted the performance on the 6MWT (β = 0.311; 95% CI 0.170, 0.453; P < 0.001), TUGT (β = -0.013; 95% CI − 0.018, − 0.008; P < 0.001) and Sit-to-Stand Test (β = 0.027; 95% CI 0.018, 0.035; P < 0.001).

**Table 4:** Univariable regression model for objective sleep measures with physical performance.

	Sleep duration (Total sleep time)		Sleep efficiency		Wake after sleep onset	
	Beta (95% CI)	*p*	Beta (95% CI)	*p*	Beta (95% CI)	*p*
**Physical functioning tests**						
6MWT	0.311 (0.170, 0.453)	< 0.001*	0.542 (− 2.898, 3.982)	0.756	− 0.595 (− 1.699, 0.509)	0.289
TUGT	− 0.013 (− 0.018, − 0.008)	< 0.001*	0.070 (− 0.050, 0.189)	0.252	0.013 (− 0.025, 0.052)	0.501
Sit-to-Stand Test	0.027 (0.018, 0.035)	< 0.001*	0.029 (− 0.187, 0.245)	0.791	− 0.043 (− 0.112, 0.027)	0.225
One-leg Standing Test	0.150 (− 0.002, 0.302)	0.052	0.237 (− 3.306, 3.780)	0.895	− 0.946 (− 2.077, 0.185)	0.101

* p < 0.05.

**Table 5:** Multivariable regression model for objective sleep measures with physical performance.

	Sleep duration^†^ (Total sleep time)		Sleep efficiency^†^		Wake after sleep onset^†^	
	Beta (95% CI)	*p*	Beta (95% CI)	*p*	Beta (95% CI)	*p*
**Physical functioning tests**						
6MWT	0.259 (0.120,0.398)	< 0.001*	2.621 (− 0.687, 5.929)	0.120	− 0.779 (− 1.816, 0.259)	0.140
TUGT	− 0.012 (− 0.017, − 0.008)	< 0.001*	0.001 (− 0.110, 0.113)	0.984	0.011 (− 0.024, 0.045)	0.552
Sit-to-stand test	0.027 (0.018 0.035)	< 0.001*	0.084 (− 0.142, 0.310)	0.463	− 0.049 (− 0.120, 0.021)	0.169
One-leg standing test	0.041 (− 0.110, 0.193)	0.589	0.827 (− 2.641, 4.295)	0.638	− 0.917 (− 1.994, 0.160)	0.095

* p < 0.05.

^**†**^Adjusted for age, gender, BMI, education level, time since diagnosis, current treatment, marital status, KPS score, step count, smoking, drinking behaviour, fatigue, anxiety, depression and Charlson Comorbidity Index.

After adjustment for covariates (i.e., background characteristics, psychological distress, fatigue, daily physical activity level, and CCI), sleep duration remained significantly associated with the 6MWT (β = 0.259; 95% CI 0.120, 0.398; P < 0.001), TUGT (β = − 0.012; 95% CI − 0.017, − 0.008; P < 0.001), and Sit-to-Stand Test (β = 0.027; 95% CI 0.018, 0.035; P < 0.001). The VIFs for all factors were below 5, indicating the absence of multicollinearity problems.

### Cut-off value of total sleep time

ROC curve analysis was performed to determine cut-off values of total sleep time for diagnosing poor performance in each of the three physical performance tests (6MWT, TUGT, and Sit-to-Stand Test). For the 6MWT, the area under the curve (AUC) was 0.689 (95% CI 0.611, 0.768; P < 0.001). The cut-off value of TST that yielded the highest Youden’s index was 257.63 min (i.e., 4.3 h). The sensitivity was 0.736 (95% CI 0.633, 0.823), specificity was 0.629 (95% CI 0.520, 0.729), positive predictive value was 0.670 (95% CI 0.569, 0.761), and negative predictive value was 0.700 (95% CI 0.587, 0.797). For TUGT, the AUC was 0.517 (95% CI 0.124, 0.910; P = 0.935). For the sit-to-stand test, the AUC was 0.680 (95% CI 0.520,0.840; P = 0.056).

## Discussion

The current study showed that shorter sleep duration as measured by actigraphy was independently associated with lower physical function as measured by the 6MWT, Sit-to-Stand Test, and Timed Up and Go Test. These associations remained even after adjustment for multiple potential confounding factors.

Our study is the first to report that shorter sleep duration as measured by actigraphy was associated with poorer physical function in cancer patients. This finding is in line with previous studies conducted among healthy and ill populations which found that sleep deprivation gives rise to more physical impairment^11,13,39^. The underlying mechanism between sleep duration and physical function remains unknown. One plausible mechanism is that sleep deprivation leads to immune system dysregulation, including a significant reduction in natural killer cell activity and an increase in pro-inflammatory cytokines^9,40^. A dysregulated immune system might be associated with a destructive metabolic profile and increased inflammatory risk^9^, which could subsequently contribute to sarcopenia, frailty, and functional decline^41^. Future research is warranted to investigate the exact underlying reasons for the physiology that link sleep duration and physical function in cancer patients. Of note, two studies conducted among a general elderly population reported that self-reported longer sleep duration is associated with greater physical function decline^42,43^. The discrepancy of the results may be attributed to the tendency of the elderly to overestimate the subjective total sleep time compared with objectively measured sleep duration. Future studies should adopt both subjective and objective measures of sleep to ensure the reliability of the sleep quality reported.

Our study revealed that subjective quality of sleep did not predict performance on the 6MWT, Sit-to-Stand Test, or One-leg Standing Test, which is in line with the results of a previous study in older adults with cancer demonstrating the insignificant association between self-reported sleep disturbance and impairment on the Short Physical Performance Battery^14^.

Interestingly, we found that the subjective quality of sleep significantly predicted TUGT. Additionally, among the physical function tests, the TUGT appears to be comparatively sensitive to reflect associations with both subjective and objective sleep measures compared to the 6MWT, Sit-to-Stand Test, and One-leg Standing test. The TUGT test encompasses numerous activity themes, namely, sit-to-stand and stand-to-sit transitions, walking, and turning, and it is often used to distinguish subjects at risk of falling^44^. Meanwhile, the other three functioning tests merely involve one activity theme: the Sit-to-Stand Test incorporates sit-to-stand and stand-to-sit transitions; the 6MWT incorporates walking and turning, and the One-leg Standing Test incorporates knee flexion^44^. It is possible that TUGT covers a wider range of physical functions and thus is more reflective of the influences exerted by poor sleep. Poor sleep is associated with greater drowsiness, poorer concentration^45^, and cognitive deficits, including abated attention and lengthened reaction time^45,46^, thereby affecting physical performance. Further studies should be conducted to study the mechanisms underlying the association between TUGT and sleep outcomes.

Concerning the performance in physical tests, our sample performed poorer in the majority of the physical functioning tests, specifically in the TUGT, Sit-to-Stand Test, and 6MWT when compared to prior studies conducted among various cancer populations. Regarding TUGT, prostate cancer patients with no distant metastasis required less time (5.2 to 7.2 s)^47^ than our sample (8.77 s). For the Sit-to-Stand Test, studies in mixed cancer types reported a range of 9 to 19 times^48–50^, while our sample completed merely 8 repetitions on average in 30 s. Regarding the 6MWT, patients with mixed cancer types walked 427 to 594 m^48,50,51^, while our sample walked 403 m in 6 min on average. Our findings suggest that advanced lung cancer patients are likely to be frailer and more vulnerable in regard to physical performance compared with other cancer populations, such as prostate, breast, and head and neck cancer. However, a comparison should be interpreted with caution considering the varied stages of cancer and study settings.

**Figure 1:**
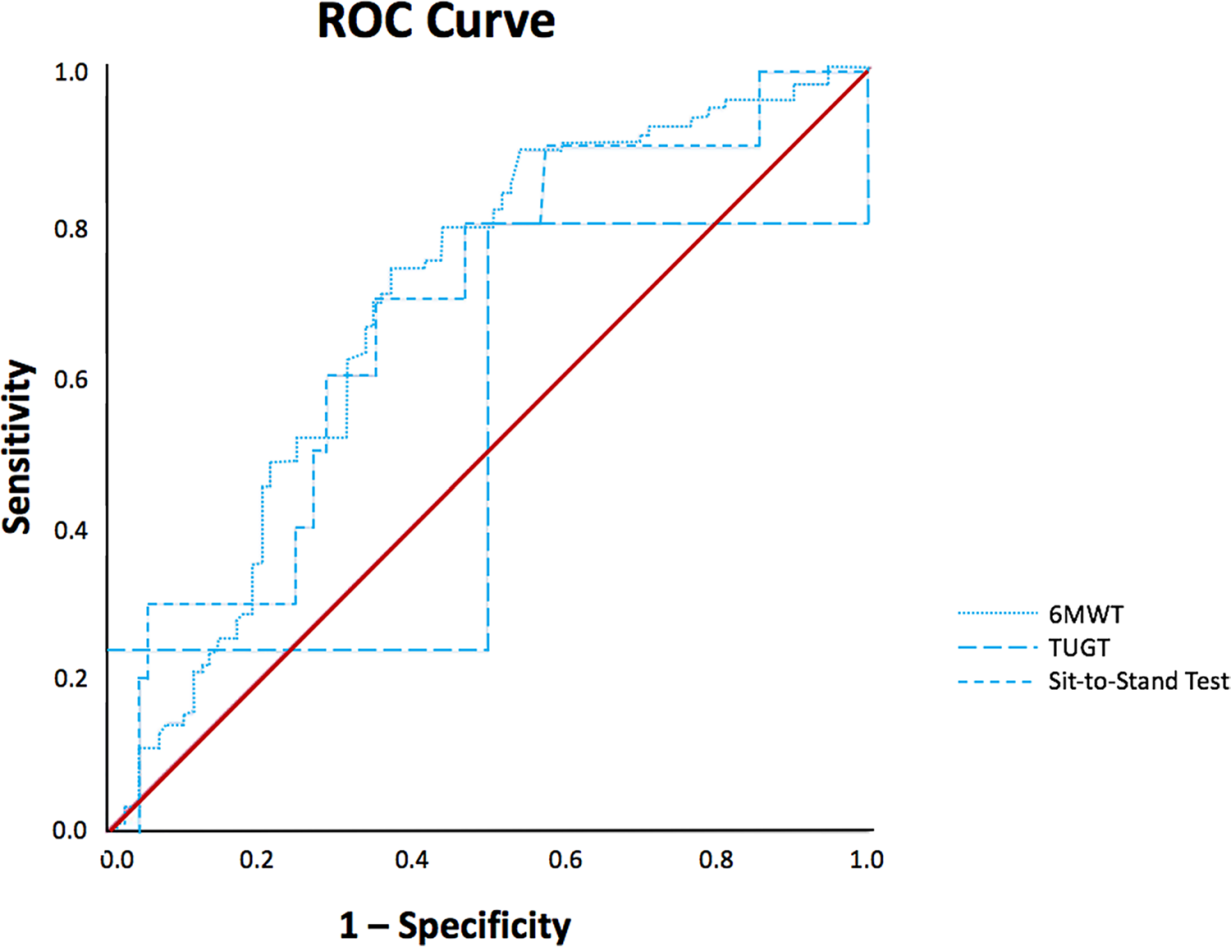
Receiver operating characteristics (ROC) curve for prediction of cut-off values of total sleep time. The area under the ROC curve (AUC) for 6MWT, TUGT and sit-to stand test were 0.689 (*P* < 0.001), 0.517 (*P* = 0.935) and 0.680 (*P* = 0.056), respectively. 6MWT: 6-Minute Walk Test; TUGT: Time Up and Go Test.

This study has several strengths. This is the first study to examine the relationship between sleep and physical performance among advanced lung cancer patients. Additionally, both subjective and objective measures of sleep were employed to assess sleep parameters, and the reliability of the results was ensured. Another strength is that the physical functioning tests performed were objective and performance-based. This study also has some limitations. First, this study was a cross-sectional study, and whether sleep duration precedes functional decline could not be determined. Second, there may be confounding factors, such as complications, chronic diseases, and pain medication use, namely opioid or psychotropics, which were not measured in our study. Furthermore, the sample size was limited and might narrow the generalizability. Future studies should include a larger sample size. Last, the optimal cut-off value of total sleep time must be validated by an independent and larger sample in the future (Fig. 1).

## Conclusions

In conclusion, our study showed that shorter sleep duration significantly predicted poorer physical performance in advanced lung cancer patients. Sleep deprivation appeared to be a significant issue that requires more attention from researchers and healthcare professionals. Intervention to ameliorate sleep deprivation is encouraged to be implemented among lung cancer patients, whilst healthcare professionals should pay more attention to the quantity of sleep in lung cancer patients, specifically for those with less than 4.3 h of sleep on average, when assessing and evaluating their condition.

## Data Availability

The datasets generated during and/or analysed during the current study are available from the corresponding author on reasonable request.
